# Chart Classification Using Siamese CNN

**DOI:** 10.3390/jimaging7110220

**Published:** 2021-10-21

**Authors:** Filip Bajić, Josip Job

**Affiliations:** 1 University of Zagreb University Computing Centre, 10000 Zagreb, Croatia; 2 Faculty of Electrical Engineering, Computer Science and Information Technology Osijek, 31000 Osijek, Croatia; josip.job@ferit.hr

**Keywords:** chart classification, chart image processing, data visualization, Siamese neural network, image processing and computer vision

## Abstract

In recovering information from the chart image, the first step should be chart type classification. Throughout history, many approaches have been used, and some of them achieve results better than others. The latest articles are using a Support Vector Machine (SVM) in combination with a Convolutional Neural Network (CNN), which achieve almost perfect results with the datasets of few thousand images per class. The datasets containing chart images are primarily synthetic and lack real-world examples. To overcome the problem of small datasets, to our knowledge, this is the first report of using Siamese CNN architecture for chart type classification. Multiple network architectures are tested, and the results of different dataset sizes are compared. The network verification is conducted using Few-shot learning (FSL). Many of described advantages of Siamese CNNs are shown in examples. In the end, we show that the Siamese CNN can work with one image per class, and a 100% average classification accuracy is achieved with 50 images per class, where the CNN achieves only average classification accuracy of 43% for the same dataset.

## 1. Introduction

In today’s world, everything can be measured and described by numbers, and the numbers can accumulate fast and create tabular data. From tabular data, it is often hard to read, notice important information, and present that information to others who may or may not have prior knowledge of it. Because of the problems mentioned above, people tend to use graphical representations of tabular data-data visualizations or chart images. Graphical representation also helps identify unusual results and compare different values, trends, and relations between different types of data. Today, the most common data visualizations (known as line, bar, and pie chart) have been used since the 18th century [1]. The majority of the used data visualizations are “locked” inside the documents, which can be digitized. These documents contain graphical and textual information linked together in one visual unit. Each year raises essential questions and issues in retrieving and storing these documents and the information that is “locked” inside. The first challenge in retrieving information from digitized data visualization images is classifying that image in one of many existing chart classes.

Chart type classification is a well-studied problem with a vast number of real-world applications dealing with chart text processing, chart data extraction, and chart description generation. Some of the existing applications include: automatic generation of a summary description of the presented chart image, exporting original data table from the chart image, adding accessibility for various screen readers, etc. Since chart image contains heterogeneous information, it is built using graphical (lines, marks, circles, rectangles, etc.) and textual (title, legend, description, etc.) components. These components are not strictly standardized, and not every component is required to be used. The designers have many choices and much freedom when designing a chart image, which often results in creating new chart classes or new chart sub-classes.

CNN can achieve state-of-the-art results in many computer vision tasks. However, this method requires many images (often 1000 or more per class) to be successful. Since new chart classes (and sub-classes) can be created daily, the number of available images is often inadequate to be used with classic CNNs. The required computing power, time to create a dataset, and teaching the network to linearly grow for each added chart class and introducing a new chart class requires retraining the network, which can be time-consuming and computationally expensive. To deal with the presented problem, and to our knowledge, we are the first to introduce the Siamese CNN in chart type classification. The Siamese network has many advantages compared to the classic CNN, about which a detailed experimental analysis will be made.

The main contributions of this paper are the Siamese CNN architecture for chart type classification, state-of-the-art results in chart type classification, and performance comparison between Siamese CNN and classic CNN.

The rest of the paper is organized in the following sections. Section 2 presents the current research on chart type classification and the usage of Siamese networks in other scientific fields. Section 3 provides brief information about Siamese CNN architecture, the used model, and used image datasets in this research. In Section 4, the verification process, the results of multiple Siamese networks, and a comparison between the classic CNN and Siamese CNN is presented. Finally, Section 5 shows the final remarks on this experiment and instructions for improvements.

## 2. Related Work

Some of the earliest scientific papers that introduced chart type classification were written in 2000 by Zhou and Tan [2,3]. These authors explained one of the most popular processes in chart type classification, which can be adopted by any method or technique. The process’s root is to create a textual and graphical separation of an input image, which outputs two images with different information. These two images are processed separately with different techniques, and the obtained results are joined for final classification. It is unnecessary to use both (textual and graphical information) for final classification, but better results can be achieved. The authors also developed a Modified Probabilistic Hough Transform for detecting lines in a bar chart. They achieved 85% average accuracy on a dataset of 35 bar chart images.

Since then, the research field of chart type classification has gained importance, and up to the day, dozens of related scientific papers have been created. Over the years, the authors have been using different key approaches for chart type classification, grouped in four categories: custom algorithm, the model-based approach, SVMs, and CNNs.

The most noted scientific papers that use custom algorithms are the Image and graphic reader [4], View [5], and Beagle [6]. In the Image and graphic reader [4], authors are using low- and high-level analysis of geometrical objects of an image. Only bar charts and pie charts are analyzed. The achieved average classification is 83% on a dataset of 55 images. In View [5] authors presented a system that automatically extracts information from a bar, pie, and line charts. Before chart classification, the geometric features of graphical components are extracted. Analyzing extracted segments, which can be filled areas or lines, the system can classify input raster images. The reported average accuracy is 97% on a dataset of 300 images. Beagle [6] is the latest work that uses a custom algorithm. It is a complete system that mines the Internet for Scalable Vector Graphics (SVG) charts, extracts them, and classifies them into 24 classes. The classification is conducted using basic statistics of SVG elements: position, dimension, number of colors, number of circles, rectangles, etc. The authors report average classification accuracy of 86%.

The model-based approach requires a model for each chart class. The model consists of specific graphical and textual features that represent a specific chart class. In the classification process, the extracted features are matched against defined models. The charts that are outside of model specification or are missing some key features will not be recognized. Mishchenko et al. describe the usage of the model-based approach [7,8]. The authors also created the area, bar, line, and pie chart models and achieved an average classification accuracy of 92%. The image corpus consisted of 980 images.

The extracted image features can be used as an input to an SVM. The basic concept of SVM is to find a hyperplane that will separate the data into two classes. The SVMs are fast when used as classifiers, but setting a hyperplane is demanding when two classes share extracted features (such as vertical bar chart and horizontal bar chart). The most noted use of SVMs is in View [5], Reverse-Engineering Visualizations [9], and Revision [10]. In Reverse-Engineering Visualization [9], authors are using SVM only for text role classification. In View [5] and Revision [10], SVMs are used for final classification based on extracted features. Revision [10] is the most cited scientific paper in this research field and also the first paper that created multi-class chart classification, including area, bar, line, map, Pareto, pie, radar, scatter, table, and Venn. The classification results of these ten chart classes are the most compared ones. The latest researches combine CNNs and SVMs. The CNNs are usually used for generating feature vectors that are used as input to an SVM. This approach is best described in VizByWiki [11], Visualizing for the Non-Visual [12], DocFigure [13], and by Kaur et al. [14]. SVMs achieve state-of-the-art results with an average classification accuracy above 90% on ten or more chart classes in the previous papers.

CNNs can also be used without SVMs and any type of image processing technique (feature extraction, pattern recognition, image segmentation, text, and graphic separation, etc.). Bajić et al. showed that CNN could achieve an average classification accuracy of 78% across ten chart classes without using image processing techniques. After applying appropriate image pre-processing techniques, as a consequence, image complexity is reduced, which increases average classification accuracy up to 89% [15]. The line and bar charts are the most researched. Kosmen et al. [16] created a CNN architecture that can classify line charts according to trend property (increasing or decreasing) and functional property (linear or exponential). The achieved average classification accuracy is 93.76%. Another work that focuses only on line charts is written by Ishihara et al. [17]. The author uses custom CNN architecture and achieves average classification accuracy of 97%. Bar charts are researched in BarChartAnalyzer [18] and by Zhou et al. [19]. BarChartAnalyzer uses CNN that classifies the bar chart into seven subtypes (simple bar, grouped bar, stacked bar, and a combination of different orientations). The average classification accuracy is 85%. In [19] authors proposed a new method for extracting textual and numerical information from bar charts. For textual information, extraction Region-based CNN combined with Tesseract Optical Character Recognition (OCR) engine is used. For numeric information, encoder-decoder is used. The achieved results are state-of-the-art for this type of architecture. The classification results also depend on the used dataset and the type of used architecture. Among the simplest CNN architectures used are LeNet (named after Yann LeCun et al. in 1989) [20], AlexNet (named after Alex Krizhevsky et al. in 2012) [21], VGG (named after Visual Geometry Group in 2014) [21], GoogLeNet (named after Google in 2014) [22], and Residual neural network (ResNet) [12]. With 1000 or more images per one chart class, the CNNs can achieve an average accuracy of 99%. The detailed comparison of AlexNet, GoogLeNet, VGG, and ResNet models is presented in Chart Decoder [22] and a brief survey written by Thiyam et al. [23]. A summary of the presented related work is summarized in Table 1. The last column, “Dataset,” is a sum of all images from different datasets that are usually split into validation, testing, and training sets. We are using the summarized value because not all authors report on all three dataset sizes. Additionally, many newer scientific papers use multiple datasets and often combinations of datasets from other authors.

## 3. The Model

The Siamese CNN was introduced by Bromley et al. for solving signature verification problems [24]. Since then, Siamese CNNs have been rarely used. The development of Compute Unified Device Architecture (CUDA) hardware and different CUDA libraries enabled fast training times. With the technology development, Siamese CNNs have become widely available. Today, they are often used in security applications, such as face recognition [25], signature validation [26], signal classification [27], speech recognition [28], etc. The Siamese CNN is a network architecture built using two or more identical (twin) networks. The main advantages and disadvantages of Siamese CNN are listed below.

The advantages of Siamese CNN:

- Can generalize to inputs and outputs that have never been seen before—a network trained on approximately ten classes can also be used on any new class that the network has never seen before, without retraining or changing any parameters;
- Shared weights—two networks with the same configuration and with the same parameters;
- Explainable results—it is easy to notice why the network responded with a high or low similarity score;
- Less overfitting—the network can work with one image per class;
- Labeled data—before training, all data must be labeled and organized;
- Pairwise learning—what makes two inputs similar;
- In terms of dataset size—less is more.

The disadvantages of Siamese CNN:

- Computationally intensive—less data but more data-pairs;
- Fine-tuning is necessary—the network layer architecture should be designed for solving a specific problem;
- Quality over quantity—the dataset must be carefully created and inspected;
- Choosing loss function—available loss functions are contrastive loss, triplet loss, magnet loss, and center loss.

Many of the listed advantages and disadvantages will be experimentally proven in the following sections.

### 3.1. The Dataset

**Figure 1:**
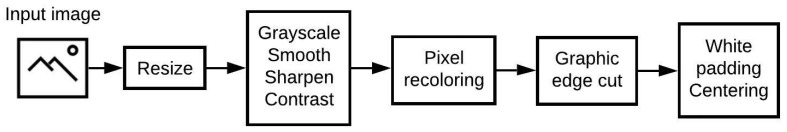
Image pre-processing algorithm.

Before explaining the datasets, image pre-processing should be noted. The pre-processing used in the creation of datasets is similar to pre-processing used in [15]. The noted “Stage 3 image processing” is fine-tuned, and the number of details on the image is further reduced. The updated algorithm is presented in Figure 1. With this algorithm, a title, coordinate axes, legend, and any additional elements on the outside of chart graphics are removed. These elements are not crucial for chart type classification based only on the shape of graphic objects used in chart creation. The images are scaled-down with a preserved aspect ratio and are normalized to 105 × 105 pixels and black-and-white color space. All images are labeled and organized as the training Siamese CNN requires true (e.g., bar and bar chart) image pairs and false (e.g., bar and line chart).

**Figure 2:**
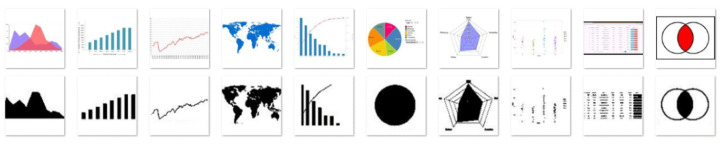
A sample from dataset 1 (3002 images), from left to right: area (308), bar (321), line (287), map (146), Pareto (228), pie (442), radar (401), scatter (288), table (403), and Venn (178). In the top row are original images, and in the bottom, images after pre-processing.

**Figure 3:**
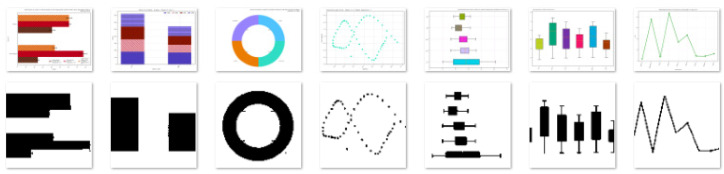
A sample from dataset 2 (198,010 images), from left to right: horizontal bar (22,304)—includes grouped and stacked, vertical bar (21,961)—includes grouped and stacked, pie (28,203)—includes pie and donut, scatter (41,703), horizontal box (21,007), vertical box (20,958), and line (41,874). In the top row are original images, and in the bottom, images after pre-processing.

In this research, three different datasets are used:

1. The dataset used in our previous research, which consists of 3002 images, is divided into ten classes, as shown in Figure 2 [15,29]. This dataset includes images collected from the Google Image search engine and ReVision system [10] (further on in the text referred to as a dataset 1).
2. International Conference on Document Analysis and Recognition (ICDAR) 2019 synthetic chart dataset, which consists of 198,010 images that are divided into seven classes as shown in Figure 3 [30] (further on in the text referred to as a dataset 2).
3. AT&T Database of Faces, which consists of 400 images, is divided into 40 classes [31].

Datasets 1 and 2 are fully pre-processed, while in dataset 3, the only applied image pre-processings are image resolution normalization and image color space normalization. Dataset 3 is only used in the Siamese CNN training because the additional 40 classes help the network to learn similarities better. Instead of this dataset, any other labeled dataset can be used. However, if no additional datasets are used, the network over-fits and the loss value oscillates between the two values. This phenomenon indicates that the model has not learned similarities.

Before training the network model, one validation set is excluded from datasets 1 and 2, which consists of 20 images per class, as listed in Table 2. This set is used as a reference point, as the images never change, and the set is never seen in the training process.

### 3.2. The Architecture

**Figure 4:**
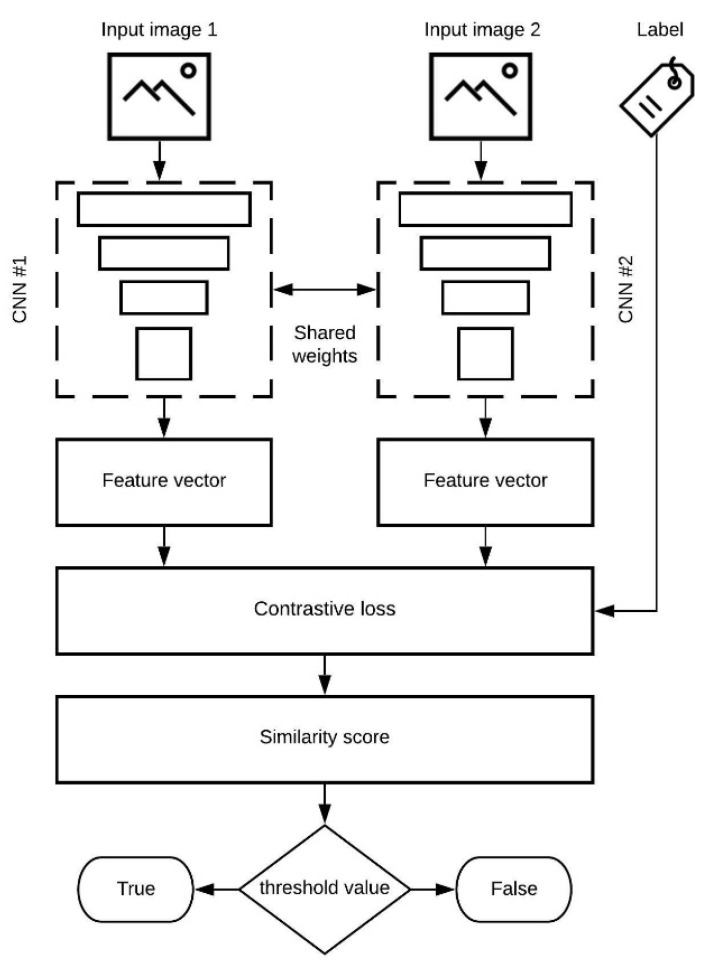
Basic concept of used the Siamese CNN model.

The presented model in Figure 4 consists of two inputs (two images), one for each CNN. The input images are pre-processed and handed to the CNN. In this research, multiple CNN architectures are tested and compared. The used CNNs are:

- Simplified VGG—the same network architecture used in our previous research. The achieved average classification accuracy over ten classes ranges from 78% to 89%, depending on the used dataset [15,29]. The achieved results are for classic CNN architecture.
- SigNet CNN—the network used for writer independent offline signature verification. The authors report accuracy ranging from 76% to 100%, depending on the used dataset [26]. The achieved results are for Siamese CNN architecture.
- Omniglot CNN—the network used on the Omniglot dataset for the validation of handwritten characters. The authors report accuracy ranging from 70% to 92%, depending on the used dataset [32]. The achieved results are for Siamese CNN architecture.

All listed network architectures were remade according to the original papers, where the authors stated details about network configuration. The input layer of the networks is reconfigured to accept new image datasets. The Siamese CNNs are identical with the same parameters, configuration, and shared weights. The parameters are mirrored and updated in both networks. Each network outputs a different feature vector. If the same input image is handed to both networks, the feature vectors will be the same. The feature vectors are used in calculating the loss function (contrastive loss), which computes a similarity score using the Euclidean distance between these two vectors. Based on the resulted similarity score and a threshold value, it can be determined if the two input images are similar and whether they belong to the same class. Used threshold values are: 0.5, 0.75, and 1. In terms of the similarity score, a lower value (closer to 0) is better (same class).

### 3.3. Experiment Setup

All CNN and Siamese CNN models were trained and tested on the Google Collab platform with PyTorch deep learning framework and enabled CUDA acceleration.

## 4. Experiments

This section summarizes the findings and contributions made. The verification process of N-way one-shot learning is described. The results of the Simplified VGG, SigNet CNN, and Omniglot CNN are compared. The detailed information of network classification results is provided, as well as a confusion table. In the end, the comparison between classic CNN and Siamese CNN is given.

### 4.1. Verification

As seen from Table 1, the CNN architecture relies on substantial data for a good outcome. Thousands of images are required for training before a network can accurately assess a new image of a chart. Newly created chart classes lack datasets, and creating and labeling a dataset is a time-consuming and expensive task. When datasets are inefficient, CNN cannot match images using learned features but can calculate similarity scores between different classes. To address this problem, FSL is used in conjunction with Siamese CNN architecture. The FSL has two main variations: Zero-shot and N-shot (or N-way-K-shot). Zero-shot learning refers to using a model to predict a class without being introduced to that class in the training process. On the other hand, N-way-K-shot is a broader concept used when the number of classes N and the number of samples K from each class is familiar.

All network models were trained from scratch using datasets described in the previous section. The used verification method is N-way one-shot learning, introduced by Lake et al. [33]. The 10-way one-shot learning is explained in Table 3. The Siamese CNN requires two input images to generate a similarity score. The input in one side of Siamese CNN is an image from the validation set (or any new image that was not used in the training process). The other CNN requires one random image from each class that was used in the training process. This creates ten image pairs for one image. Comparing each image pair, the Siamese CNN calculates a similarity score.

The expected highest similarity, i.e., similarity score closest to 0, according to Table 3, should be SS3. If SS3 is the lowest value in the group and within the set threshold value, this is treated as a correct classification (same class); otherwise, this is incorrect. Repeating the algorithm *x* times, the class accuracy *CA* is calculated. In Equation (1), *CC* represents the number of correct classifications within a class.

(1) $$CA=\frac{100\times CC}{x}\;\left[\%\right]$$

For verification, a set of 20 images per class (*x* = 20) is used. With this method, 200 image pairs are tested for one class or 2000 image pairs for ten chart types.

In this algorithm, the similarity score depends on two random variables, the input image 1 (new image) and the input image 2 (random training image). To eliminate one random variable (random training image), the input image is tested against all trained images from each class. The highest similarity images from each class are grouped, and new image pairs for verification are created. With this method, 4000 image pairs are tested for one class or 40,000 image pairs for ten chart types.

### 4.2. Results

To validate the performance of the proposed architecture, a set of experiments is conducted. The goal is to evaluate the performance using all three models and determine which model achieves the highest average classification accuracy on chart images.

Table 4 shows the 10-type average classification accuracy that was conducted using dataset 1. The three used networks were trained from scratch. Planned comparisons revealed that the Simplified VGG outperforms the Omniglot CNN and SigNet CNN. When used as the Siamese CNN, the Simplified VGG achieves results similar to our previous work, used as a classic CNN. It must be pointed out that the results are achieved using less than 10% of the total images from dataset 1. The two other networks achieve around 50% worse results than reported in papers. The reason is network layer construction. The Simplified VGG is a network that is adapted specially for chart-type classification. When the input image passes through the network layers, the image is segmented into smaller sub-images. The Omniglot CNN and SigNet CNN are specially designed for searching and learning imperfections of two images on a pixel level, while Simplified VGG observes the image as a whole. Since the input images are heavily pre-processed, they contain image noise that the Omniglot CNN and SigNet CNN are detecting.

**Figure 5:**
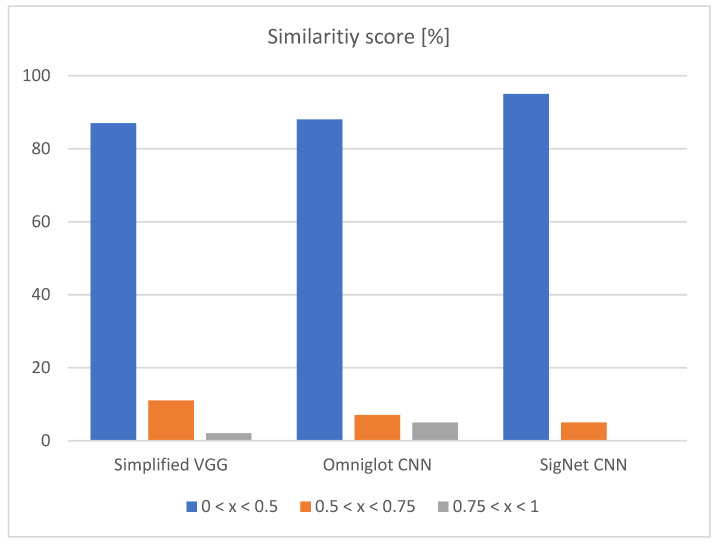
Input vs. random train image from each class—similarity score.

**Figure 6:**
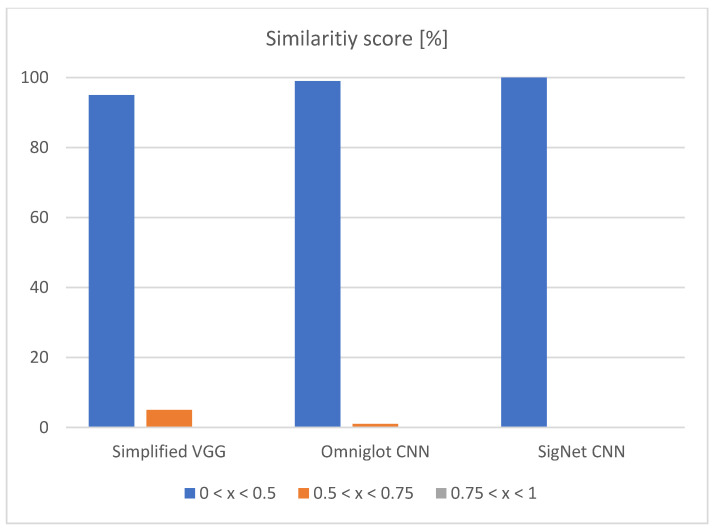
Input vs. highest similarity image from each class—similarity score.

From the left side of Table 4, it can be seen that choosing a random train image for comparison can have a hit-or-miss result. If the system chooses the correct image, the classification result can be 100% and 0% if the image is not similar. To avoid this phenomenon, the quality of the dataset is more important than the quantity of the dataset. On the right side of the table, 20 times more image pairs are used, which increases the average classification accuracy by 15%. The difference between these two approaches can also be seen in Figure 5 and Figure 6.

In both approaches, the highest amount of correctly classified images is between 0 and 0.5. This confirms that all three networks are correctly trained, and they are confident in the results they give. When using the higher amount of image pairs, the third column (0.75 < *x* < 1) is eliminated, as shown in Figure 6.

For statistical comparison of the models, a statistical hypothesis test is conducted using McNemar’s test. A McNemar’s test uses a contingency table, which is a 2 × 2 table that contains binary variables as correct or incorrect. Each model’s prediction on the same image is noted as both models predicted correct or incorrect, or only one model correctly predicted. This test calculates whether the two models disagree in the same way or not. In Table 5, all models’ *p*-values are compared against a significance level of 0.05. In all cases, the *p*-value is less than 0.05, and the null hypothesis H0 is rejected. The rejected H0 shows a significant difference in the disagreements between the models, and we conclude that the models make considerably different predictions when introduced to the same images.

Since slightly superior results are achieved with Simplified VGG, additional information is presented in the confusion table, Table 6. The horizontal rows represent known (seen) classes, and the vertical columns represent predicted classes. The number of correct predictions is displayed in a diagonal green row (maximum is 20). Red-colored cells show the number of wrong predictions. The Siamese CNN can also be used to classify chart types that were not used in the training process; therefore, the network does not know of them. To prove this statement, a box plot from dataset 2 is used. When letting the network choose a random image pair, the results are slightly worse than with seen classes during the training. When the network uses all available image pairs, the results are the same as for the seen classes during the training.

The average classification accuracy between 10-type and 11-type slightly decreases, which is expected when the number of types for classification increases.

To compare the classic CNN with the Siamese CNN, additional tests are created for the Simplified VGG. The network is trained 16 times from scratch (eight times as a classic CNN and eight times as Siamese CNN). The network configuration and the training parameters were always the same. The training is conducted using dataset 2 (7—type chart classification). The same batch of images is used when training classic CNN and Siamese CNN. The goal of training each network from scratch eight times is to find the minimal training dataset size to achieve state-of-the-art results. In Table 7, e.g., “t10” referees to training dataset with ten images per class. For the model, verification is always used the same set of images, validation set. Verification of Siamese CNN is conducted by creating image pairs for 7-way-one-shot learning. Comparing the results from Table 7 shows how the number of images and image pairs impacts classification accuracy and the required time for classification. The classic CNN does not require the pairing of input images with training images, which makes it equally fast with any size of the training dataset. However, even with 500 images per class, the average classification accuracy did not reach 100%. This type of CNN is not usable with small training datasets, and competitive results start showing when the number of images per class reaches 200 or more. On the other hand, the Siamese CNN can work with one image per class.

**Figure 7:**
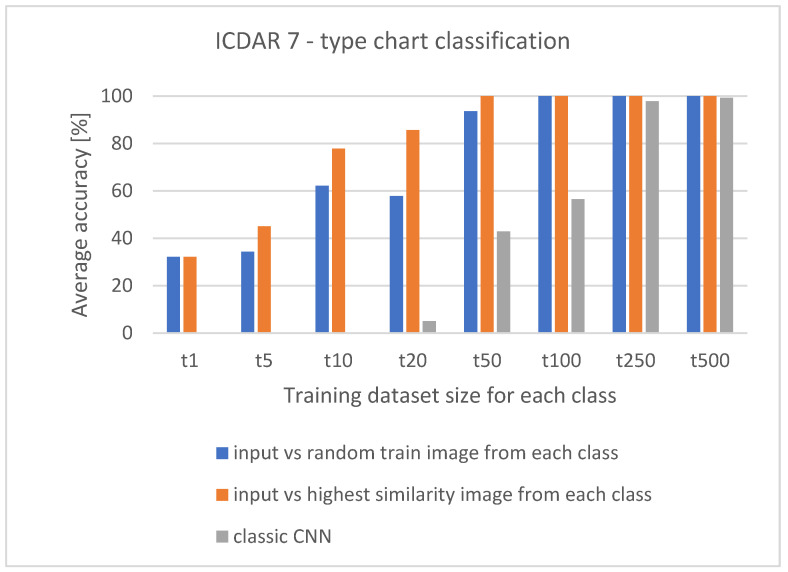
The summary of the average classification accuracy from Table 8. Between t10 and t20, the hit-or-miss effect can be seen. The state-of-the-art results for Siamese CNN are achieved with 50 training images per class.

The competitive results are achieved with the datasets between 20 and 50 images per class, and state-of-the-art results are achieved with just 50 images per class. The average classification and F-1 score should constantly increase if the number of images is also increasing. Although this is true, it is false when pairing the input image with a random train image. In Figure 7, the effect of the hit-or-miss random image can be seen between “t10” and “t20,” where average classification accuracy decreases.

For statistical comparison, the same McNemar test is conducted as for Table 5. When comparing two Siamese CNNs, the significant difference can only be seen between “t5” and “t50,” and H0 can be rejected. This is expected behavior since one Siamese CNN is using random train images for generating similarity scores. When the Siamese CNNs are compared to classic CNN, the H0 can be rejected up to “t100”, as shown in Table 8. This confirms that these models make considerably different predictions that are in accordance with average classification accuracy and F-1 score from Table 7.

## 5. Conclusions

This paper focuses on the classification of chart images using the Siamese CNN, which has never been conducted before. This work is motivated by the lack of publicly available datasets and a continually growing number of chart types. The conducted research proves that Siamese CNN can be used with chart type classification. The results of three used Siamese CNN architectures show that the network layer construction impacts classification results. Regarding N-way-one-shot learning, choosing image pairs can have a hit-or-miss result, which indicates the quality over quantity of the used dataset. When compared to a classic CNN, the Siamese CNN outperforms the required image dataset size and achieved an average classification accuracy and F-1 score. We have shown that the Siamese CNN can also generalize the input never seen before and achieve competitive results. When trained on seven chart types, the Siamese CNN achieved state-of-the-art results, which is 100% average classification accuracy and F-1 score.

In the future, other loss functions (triplet loss, magnet loss, center loss) will be tested and compared. The plan is also to increase the number of chart types to 20 or more. The image pre-processing algorithm can be further optimized, and the number of details in the image can be further decreased, resulting in achieving 100% accuracy with an even lower number of images per class.

## Figures and Tables

**Table 1:** A short summary of a presented related work.

Ref.	Year	Method	Results CA—Classification Accuracy DE—Data Extraction	Dataset
[2]	2000	Modified Probabilistic Hough Transform	Bar reconstruction rate 92.30%. Correlation of bar pattern and text 87.30%	20
[3]	2000	Modified Probabilistic Hough Transform	CA: synthetic bar 90.00%, real images of a bar 87.30%, hand-draw bar 78.00%	35
[4]	2001	Custom algorithm	CA: bar 73.00%, pie 93.00%	55
[7]	2011	Model-based	CA: line 100.00%, area 96.00%, bar 96.00%, 2D pie 74.00%, 3D pie 85.00%	980
[8]	2011	Model-based	CA: line 100.00%, area 96.00%, bar 96.00%, 2D pie 74.00%, 3D pie 85.00%	980
[10]	2011	SVM	CA: area 88.00%, bar 78.00, line 73.00%, map 84.00%, Pareto 85.00%, pie 79.00%, radar 88.00%, scatter 79.00%, Table 86.00%, Venn 75.00%.	2601
[5]	2012	Custom algorithm, SVM	CA: 97.00% (bar, pie, line)	300
[9]	2017	CNN, SVM	CA: area 95.00%, bar 97.00%, line 94.00%, map 96.00%, Pareto 89.00%, pie 98.00%, radar 93.00%, scatter 92.00%, Table 98.00%, Venn 91.00%	5125
[20]	2017	CNN	CA: 70.00% (area, bar, line, map, Pareto, pie, radar, scatter, table, Venn)	4837
[6]	2018	Custom algorithm	CA: from 83.10% to 94.00%	33,778
[11]	2018	CNN	CA: non-dataviz 89.00%, dataviz 93.00%	755
[22]	2018	CNN	CA: 99%. (bar, pie, line, scatter, radar) DE: from 77.00% to 89.00%.	11,174
[12]	2019	CNN	CA: area 96.00%, bar 98.00%, line 99.00%, map 97.00%, Pareto 100.00%, pie 96.00%, radar 94.00%, scatter 98.00%, Table 92.00%, Venn 97.00%. DE: 87.67%.	2398
[13]	2019	CNN	CA: 28 types from 88.96% to 92.90%	33,000
[14]	2020	CNN	CA: ordination 98.00%, map 97.00%, scatter 89.00%, line 91.00%, dendogram 97.00%, column 97.00%, heat map 95.00%, box 96.00%, area 80.00%, network 91.00%, histogram 83.00%, timeseries 84.00%, pie 97.00%, stack area 96.00%	4073
[15]	2020	CNN	Comparison of image preprocessing methods to overall chart classification	3002
[16]	2020	CNN	CA: line 93.75%	1920
[17]	2020	CNN	CA: line 97.00%	4718
[21]	2020	CNN	Comparison of pre-trained and fine-tuned models	4249
[18]	2021	CNN	CA: bar 85%	1400
[19]	2021	CNN	DE: bar 85%	30,480
[23]	2021	CNN	Comparison of deep learning models	57,097

Although multiple CNNs can be used for different classification tasks, none of the authors used Siamese CNN for chart-type classification.

**Table 2:** The total number of images in each dataset after excluding the validation set.

Dataset 1		
Chart type	Available	Validation Set
Area	288	20
Bar	301	20
Line	267	20
Map	126	20
Pareto	208	20
Pie	422	20
Radar	381	20
Scatter	268	20
Table	383	20
Venn	158	20
Total	2802	200
	3002	
Dataset 2		
Chart type	Available	Validation Set
Pie	28,183	20
Line	41,854	20
Scatter	41,683	20
Horizontal box	20,987	20
Vertical box	20,938	20
Horizontal bar	22,284	20
Vertical bar	21,941	20
Total	197,870	140
	198,010	

**Table 3:** Example of a 10-way one-shot learning. The highest expected similarity score should be SS3.

Image Pair (Class N)	Input Image 1 (New Image)	Input Image 2 (Known Image)	Similarity Score (SS)
1	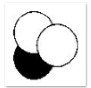	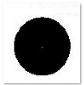	SS1
2	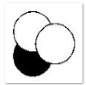	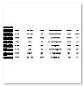	SS2
3	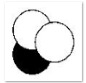	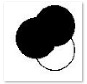	SS3
4	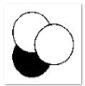	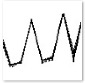	SS4
5	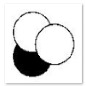	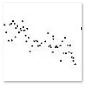	SS5
6	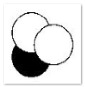	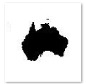	SS6
7	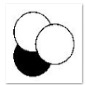	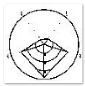	SS7
8	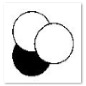	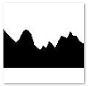	SS8
9	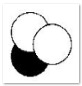	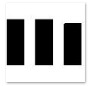	SS9
10	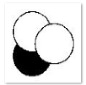	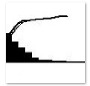	SS10

**Table 4:** Ten-type average classification accuracy. The testing of each architecture is conducted on the same validation set from Table 2. In terms of average accuracy and F-1 score, the Simplified VGG outperforms other networks.

	Input vs. Random Train Image from Each Class			Input vs. Highest Similarity Image from Each Class		
	Simplified VGG	Omniglot CNN	SigNet CNN	Simplified VGG	Omniglot CNN	SigNet CNN
Accuracy (%)	68.00	40.00	30.00	82.00	63.00	41.00
Precision (%)	68.00	39.50	29.50	81.50	63.00	40.50
Recall (%)	69.36	46.32	29.83	84.24	66.67	42.34
F-1 score (%)	67.86	39.68	29.43	81.94	63.00	40.54

**Table 5:** The McNemar’s test with a significance level of 0.05. All model comparisons rejected null hypothesis H0.

	*p* < 0.05	H0
input vs. random train image from each class		
Simplified VGG—Omniglot CNN	true	reject
Simplified VGG—SigNet CNN	true	reject
Omniglot CNN—SigNet CNN	true	reject
input vs. highest similaritiy image from each class		
Simplified VGG—Omniglot CNN	true	reject
Simplified VGG—SigNet CNN	true	reject
Omniglot CNN—SigNet CNN	true	reject

**Table 6:** There are two cases in a table: (a) input vs. random train image from each class; (b) input vs. highest similarity image from each class (shown in parenthesis). Each class can have a maximum of 20 correct predictions.

Simplified VGG	Seen Classes during Network Training										Unseen Class	Accuracy (%)
	Area	Bar	Line	Map	Pareto	Pie	Radar	Scatter	Table	Venn	Box	
Area	15 **(16)**	5 **(4)**	-	-	-	-	-	-	-	-	-	75 **(80)**
Bar	-	15 **(16)**	-	-	5 **(4)**	-	-	-	-	-	-	75 **(80)**
Line	-	-	16 **(16)**	-	-	-	-	4 **(4)**	-	-	-	80 **(80)**
Map	5 **(6)**	-	-	10 **(14)**	-	-	-	-	-	5 **(0)**	-	50 **(70)**
Pareto	-	3 **(3)**	2 **(0)**	-	15 **(17)**	-	-	-	-	-	-	75 **(85)**
Pie	-	-	-	-	-	17 **(20)**	-	-	-	3 **(0)**	-	85 **(100)**
Radar	-	-	4 **(4)**	-	-	-	13 **(16)**	3 **(0)**	-	-	-	65 **(80)**
Scatter	-	-	3 **(4)**	-	-	-	4 **(0)**	11 **(16)**	2 **(0)**	-	-	55 **(80)**
Table	-	-	-	-	-	-	5 **(0)**	3 **(6)**	12 **(14)**	-	-	60 **(70)**
Venn	-	-	-	2 **(0)**	-	6 **(2)**	-	-	-	12 **(18)**	-	60 **(90)**
Box	-	-	4 **(2)**	8 **(0)**	-	-	4 **(2)**	-	-	-	4 **(16)**	20 **(80)**
(a) input vs. random train image from each class												
10—type classification (without box plot) average:					**Precision**	68.00%	**Recall**	69.36%	**F-1 score**	67.86%	**Accuracy**	68.00%
11—type classification (with box plot) average:					**Precision**	63.64%	**Recall**	67.49%	**F-1 score**	62.57%	**Accuracy**	64.00%
(b) input vs. highest similarity image from each class												
10—type classification (without box plot) average:					**Precision**	81.50%	**Recall**	84.24%	**F-1 score**	81.94%	**Accuracy**	82.00%
11—type classification (with box plot) average:					**Precision**	81.36%	**Recall**	84.19%	**F-1 score**	81.86%	**Accuracy**	81.00%

**Table 7:** Comparison of Siamese CNN and classic CNN conducted on dataset 2 (7-type chart classification). Both networks use the same dataset for training and verification. Regarding the average classification accuracy and F-1 score, when small datasets are used, Siamese CNN outperforms classic CNN. When comparing the required time for image classification, classic CNN is the fastest.

Dataset	Simplified VGG as Siamese CNN												Simplified VGG as Classic CNN				
	Input vs. Random Image from Each Class						Input vs. Highest Similarity Image from Each Class										
	Image pairs	Time to classify	Accuracy (%)	Precision (%)	Recall (%)	F-1 score (%)	Image pairs (s)	Time to classify	Accuracy (%)	Precision (%)	Recall (%)	F-1 score (%)	Time to classify	Accuracy (%)	Precision (%)	Recall (%)	F-1 score (%)
t1	140	2 s	32.14	32.14	40.46	32.45	140	2 s	32.14	32.14	40.46	32.45	1 s	0	0	0	0
t5	140	2 s	34.28	34.28	41.47	35.36	700	7 s	45.00	45.00	49.30	46.10	1 s	0	0	0	0
t10	140	2 s	62.14	62.14	64.39	62.53	1400	15 s	77.85	77.85	78.91	78.66	1 s	0	0	0	0
t20	140	2 s	57.85	57.85	60.65	57.45	2800	30 s	85.71	85.71	86.60	85.62	1 s	5.00	5.00	15.99	5.85
t50	140	2 s	93.57	93.57	94.28	94.28	7000	1 m	100	100	100	100	1 s	42.85	42.85	48.22	44.87
t100	140	2 s	100	100	100	100	14,000	2 m	100	100	100	100	1 s	56.42	56.42	57.70	56.63
t250	140	2 s	100	100	100	100	35,000	5 m	100	100	100	100	1 s	97.85	97.85	98.70	98.56
t500	140	2 s	100	100	100	100	70,000	10 m	100	100	100	100	1 s	99.28	99.28	99.32	99.28

**Table 8:** The McNemar’s test with a significance level of 0.05. The results are in accordance with the classification results from Table 7.

Dataset	Input vs. Random Image from Each Class—Input vs. Highest Similarity Image from Each Class		Input vs. Random Image from Each Class—Classic CNN		Input vs. Highest Similarity Image from Each Class—Classic CNN	
	*p* < 0.05	H0	*p* < 0.05	H0	*p* < 0.05	H0
t1	false	failed	true	reject	true	reject
t5	true	reject	true	reject	true	reject
t10	true	reject	true	reject	true	reject
t20	true	reject	true	reject	true	reject
t50	true	reject	true	reject	true	reject
t100	false	failed	true	reject	true	reject
t250	false	failed	false	failed	false	failed
t500	false	failed	false	failed	false	failed

## Data Availability

Not applicable.
